# Challenges in measuring depression among Ugandan fisherfolk: a psychometric assessment of the Luganda version of the Center for Epidemiologic Studies Depression Scale (CES-D)

**DOI:** 10.1186/s12888-020-2463-2

**Published:** 2020-02-05

**Authors:** Amanda P. Miller, Michael Kintu, Susan M. Kiene

**Affiliations:** 1 Division of Infectious Disease and Global Public Health, University of California, San Diego, La Jolla, CA USA; 2 Division of Epidemiology and Biostatistics, San Diego State University School of Public Health, 5500 Campanile Drive (MC-4162), San Diego, CA 92182 USA; 3 Wakiso Integrated Rural Development Association, Entebbe, Uganda; 4 Alcohol Research Center of HIV, Brown University School of Public Health, Providence, RI USA

**Keywords:** Depression, HIV, CES-D scale, Uganda, Fisherfolk, sub-Saharan Africa

## Abstract

**Background:**

Depression is a prevalent and serious mood disorder and a major source of disability adjusted life years (DALY) in Uganda. Furthermore, evidence from Uganda and other countries throughout sub-Saharan Africa suggests that nearly a third of persons living with human immunodeficiency virus (HIV) suffer from depression and it adversely affects healthcare seeking behavior. The high burden of disease attributable to depression makes data on the prevalence of depression in Uganda, a country with a generalized HIV epidemic, a public health priority. This paper describes the psychometric properties of the Center for Epidemiologic Studies-Depression (CES-D) measure when administered to men and women residing in three fishing communities along the shore of Lake Victoria.

**Methods:**

We applied methods based on item response theory and classical test theory approaches to assess individual item characteristics, conducted exploratory factor analysis and assessed internal reliability, and construct and content validity of the measure. All analyses were performed in R Studio.

**Results:**

The study sample consisted of 300 residents of fishing communities in Wakiso District, Uganda. Fifty-six percent of the sample was female and 19.7% reported being HIV positive. Seven items of the measure that did not perform well, either because they could not differentiate between levels of the latent trait or because they did not map onto the primary factor, were removed from the scale. A single factor structure best fit our final set of 13-items and we found an overall coefficient alpha of 0.89, indicating high internal consistency in this population.

**Conclusions:**

Based on our findings, we recommend that future use of the CES-D in this population utilize our revised scale with the final set of 13-items. The addition of other measures that can improve the rigor of CES-D validation efforts, such as inclusion of a clinical depression measure and administration in both a clinical and a general population sample in this setting are needed.

## Background

Depression, clinically referred to as major depressive disorder (MDD), is a prevalent and serious mood disorder that effects more than 300 million people (~ 4% of the population) globally [1]. Depression is caused by a combination of biological, behavioral, psychological and environmental factors; individuals with a family history of depression, experiencing a traumatic life event, or living with a chronic illness are at increased risk [2]. Manifestation of depressive symptoms differ in severity from person to person and not all individuals experience the same depressive symptoms. Prior research in Uganda suggests that depression is a risk factor for human immunodeficiency virus (HIV) in this setting and commonly co-occurs with other HIV risk factors such as alcohol and other substance use and intimate partner violence [3].

Evidence from studies in Uganda and elsewhere in sub-Saharan Africa suggest that depression is common among persons living with HIV (PLWH) [4]; A recent meta-analysis found a pooled depression prevalence of 31% among PLWH in Uganda, which is nearly ten times higher than prevalence estimates in the general population (3.35%) [5, 6]. There is also evidence that depression adversely affects healthcare seeking behavior, including engagement in HIV care, treatment adherence, and key clinical outcomes such as CD4 count and HIV viral load [5, 7–10]. In Uganda, which faces a generalized HIV epidemic (national prevalence of 6.7% with hotspot prevalence in the fishing communities approaching 42%), the impact of treatment non-adherence on the National HIV/AIDS response can be far reaching, as non-adherence to antiretrovirals precludes viral suppression [11, 12]. Viral suppression is critical to both HIV prevention and treatment efforts because an individual who is virally suppressed will not only have better treatment outcomes, they will also be at very low risk of transmitting the HIV virus to another individual [13–15]. Depression is also a significant public health issue in its own right.

In 2017, the Global Burden of Diseases study found that the leading cause of years lived with a disability (YLD) in Uganda was mental health disorders (16.01% of all YLDs) [16]. Depression is also a major source of disability adjusted life years (DALY), contributing 234,939.61 DALYs in 2017, which accounted for 1.4% of the total disease burden in Uganda that year [16]. Given the heavy burden of disease attributable to depression as well as the role depression plays in exacerbating other co-occurring health conditions, accurate estimates of depression prevalence are critical for effective public health planning in this setting. A number of tools that can be self-administered or administered by lay individuals exist for the purposes of data collection and screening for depressive symptomology [17–20]. One of the most commonly used depression scales, especially among PLWH, is the Center for Epidemiologic Studies Depression scale (CES-D) [19].

### Rationale for assessing the psychometric properties of the CES-D in Uganda

Since its development, the full 20-item CES-D has been translated into many languages and used in a wide array of settings globally [21]. In sub-Saharan Africa, it has been translated and validated in specific populations (e.g. PLWH, pregnant women) in Zambia, South Africa and Uganda [22–24]. In Uganda, four prior studies have used the CES-D to screen for depression [23, 25–27], but only one of these studies assessed and reported the reliability and validity of the scale [23]. Natamba et al. used the CES-D to screen for depression among a small sample of pregnant women attending an antenatal clinic (ANC) at Gulu Regional Referral Hospital in Northern Uganda (*n* = 123) [23]. For the study, the CES-D was translated into the two local Luo languages: Acholi and Langi. The authors found that the CES-D had very high internal consistency in this population (Cronbach’s alpha = 0.92), indicating participants had high consistency in responses across items. They also assessed criterion validity by comparing CES-D scores with participants’ scores on the Mini-International Neuropsychiatric Interview (MINI-D), a clinician administered tool used to diagnose depression. In this population, the CES-D had good diagnostic efficiency in detecting MINI-defined current MDDs (AUROC = 0.82). Severity of depression differed significantly by HIV serostatus, with women living with HIV in the study scoring an average of 6.2 points higher on the CES-D than HIV-uninfected women [23]. Based on their findings, the authors suggested that a score of 17 was an optimal cut off for the identification of MDD using the CES-D in this population.

While Natamba et al’s. findings are a helpful addition to a sparse evidence base regarding the performance of translated depression screening measures in Uganda, Acholi and Langi, are only spoken in Northern Uganda, limiting the usefulness of this translated scale elsewhere in Uganda. The present paper describes the psychometric properties of the Luganda version of CES-D using data collected from a sample of men and women residing in fishing communities along Lake Victoria. We applied methods based in item response theory (IRT) and classical test theory (CTT) approaches to assess individual item characteristics; conducted exploratory factor analysis; and assessed internal reliability, and content and construct validity of the CES-D. The generalizability of our findings will be limited to settings with similar characteristics to the fishing communities included in this analysis, but Luganda is the most widely spoken language in the country (spoken by 19% of Ugandans) and it is spoken in fishing communities across multiple districts (e.g. Rakai, Wakiso) [28]. Our findings may also be generalizable to other populations in Uganda that are considered most-at risk populations, such as sex workers working in places other than the fishing communities and truck drivers. Both of these populations experience a high prevalence of HIV and heavy alcohol use. Furthermore, although findings from scale validation efforts are limited in their external validity, the improved internal validity that such an assessment can provide is critical to public health efforts to accurately estimate the burden of depression in this setting, measure trends and ultimately, identify populations to target for intervention and funding allocation.

## Methods

### Study population and setting

The data included in this paper were collected as part of a parent study which examined how alcohol and experience of problems related to alcohol use (dependence, physical, psychological and social harms) are associated with HIV risk behavior and HIV status in fishing communities in Uganda. The cross-sectional study was conducted in three communities along the shores of Lake Victoria in Wakiso District, Uganda: Kasenyi, Bussi Island, and Burgiri. The primary source of income in these communities is generated through the fishing industry (fishing, processing fish, selling fish, etc.) as well as services supporting the local population (sex work, bars, restaurants, and small shops) and most individuals in the community identify as fisherfolk. Fishing communities along Lake Victoria have high prevalence of HIV, heavy alcohol use, and intimate partner violence. Participants were recruited via quota-based snowball sampling from four professions in these communities that comprise most of the employment opportunities: fisherman, fishmongers, alcohol vendors and commercial sex workers. Equal numbers of participants were recruited from the four professional strata. Additional eligibility criteria included being at least eighteen years of age and providing informed consent. The present study was the first to look at depression in this context (fishing communities) and population (fisherfolk) in Uganda so no a priori differences in severity of depression between the four professions was assumed. This assumption is supported by some of our prior qualitative work in another fishing community in Wakiso district which found that individuals engaged in these occupations have a number of similarities, including living in the social and physical environment of fishing communities and being driven to migrate from their home communities to the fishing communities for the same reason: economic desperation due to a lack of education and a lack of employment opportunities in their home communities [29, 30]. Community mobilization and participant recruitment were facilitated through a collaboration with the Wakiso Beach Management Units (BMUs) which are comprised of trusted local leaders in the communities. A more detailed description of the study population as well as the data collection methods can be found in prior publications [31, 32].

### Data collection methods

All data were collected in-person through an interviewer-administered questionnaire. Interviews typically lasted one hour, and data was captured electronically using computer-assisted personal interviewing (CAPI) software. The questionnaire was developed in English and then translated by an experienced Luganda translator and administered in Luganda, the local language. Translations were reviewed and verified by two native Luganda-speaking research staff and discrepancies were resolved through discussion. Participants received 10,000 Shillings ($3 US dollars) as compensation for their participation.

### Measures

#### Sociodemographic variables and HIV status

Gender, HIV status, and monthly earnings were self-reported by participants as part of the questionnaire. If an individual did not know their HIV status or reported being HIV negative but had not received an HIV test in over a year, their status was considered ‘unknown’.

#### Depression measure

The Center for Epidemiologic Studies Depression (CES-D) scale was used to assess depressive symptoms. The scale contains 20-items, each with four response options and assesses symptoms over the past seven days: Rarely or none of the time (less than 1 day); Some or a little of the time (1–2 days); Occasionally or a moderate amount of time (3–4 days); and Most or all of the time (5–7 days) [19]. Initial assessment of the psychometric properties of this scale in the original validation study revealed a four factor structure and a cut off score for probable depression of 16 [19].

### Statistical methods

All analysis was conducted using R Studio [33]. The dataset was cleaned and imported into R Studio. Scoring for reverse coded items from the CES-D (items 4, 8, 12 and 16) were reversed and items were summed to capture total CES-D score. To assess unidimensionalilty and explore factor structure, we performed an exploratory factor analysis (EFA). As determined a priori, four criteria were established for analyzing and interpreting the results of the factor analysis procedures: (1) eigen values greater than one, (2) the proportion of the variance accounted for by the factors (> 0.3) (3) no significant crossloading of items onto multiple factors (> 0.3) and (4) each factor having a minimum of three items. To identify the number of underlying factors, parallel analysis was used. Factor rotation was performed using promax non-orthogonal (oblique) rotation to increase interpretability. Since the introduction of the CES-D in 1977, researchers have identified over twenty different factor structures using the CES-D, none exceeding a four factor solution [34]. For this reason, we predicted that the factor structure in our sample would not exceed four factors.

Reliability of the final scale was assessed by first completing EFA. After the number of factors present in this sample was determined, items not loading highly onto the primary dimension or not meeting our factor inclusion criteria were dropped. Next, nonparametric and parametric item response model approaches were applied to look at how different items in the scale performed. To see how well the CES-D could estimate the latent trait (depression) across abilities (the specritum of depression severity) we generated a test information function. Finally, Cronbach’s coefficient alpha and McDonald’s coefficient omega for the final scale were calculated [35, 36]. Chronbach’s alpha is often considered a conservative, lower bound estimate of reliability. We know that alpha (i) can be inflated by the number of items in the scale and sample size; (ii) can be increased through the inclusion of redundant items; and (iii) assumes essential tau equivalency, meaning it assumes roughly equal covariance of each scale item with the latent variable, so we also calculated McDonald’s coefficient omega [37]. Content validity was assessed by grouping scale items by domain to assess domain representation after items were removed during the EFA. Construct validity was assessed by comparing item performance (item ranking) within each of the domains to a reference sample.

## Results

### Descriptive characteristics of sample and scale

**Fig. 1:**
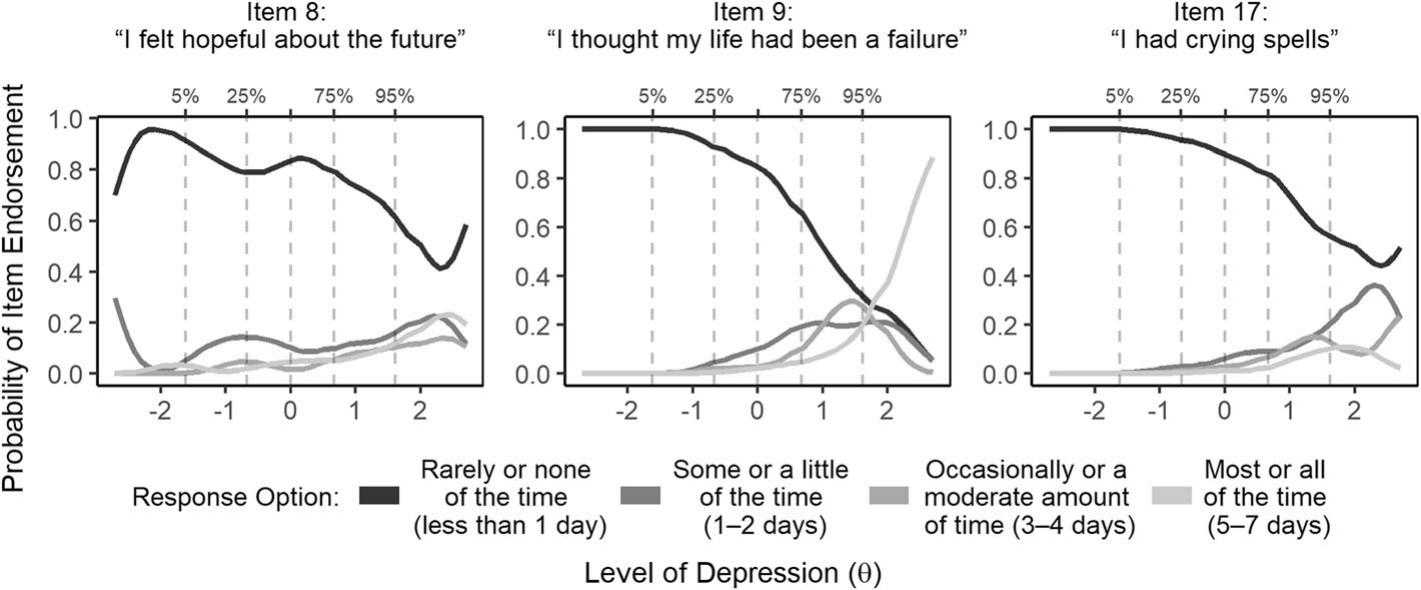
Option Characteristic Curves (OCCs) for items removed prior to exploratory factor analysis. The Y axis indicates the probability of selecting a particular response option and the X axis represents levels of the latent construct. The plotted lines represented the different response options and the dotted vertical lines within the plot areas indicate the 5th, 25th, 50th, 75th, and 95th quintiles

A total of 300 persons participated in the study. Descriptive characteristics of the study sample can be found in Table 1 and are further described in Kiene et al. [31]. There was substantial variability in reported monthly earnings; the distribution of earnings was skewed right with the majority of participants reporting lower incomes and a few participants reporting much higher incomes. All 300 participants provided responses to each of the 20 CES-D scale items. Out of the range of possible scores (0–60), participant CES-D scores for the full scale ranged from 0 to 39 with a mean score of 11.4 (standard deviation (SD) 8.71). Table 2 contains the proportion of respondents who selected each response option for every item. First, we looked at the option characteristic curve (OCC) for each item to determine which items were performing well (i.e. providing unique information about levels of depression and effective shifts to higher response options as levels of the trait increased) and which were not providing us with sufficient information about the latent trait. Items 8,9 and 17 demonstrated little to no variability (see Table 2) in responses across respondents, with the majority (79, 76 and 86%, respectively) of respondents endorsing response option zero, “rarely or never”, and low endorsement frequency across the other response options. See Fig. 1 for the OCCs of these three items.

**Table 1:** Descriptive characteristics of study sample

Characteristic	*N* = 300 (%)
Gender	
Men	132 (44.0%)
Women	168 (56.0%)
Marital Status	
Currently Married	129 (43.0%)
Divorced	128 (42.7%)
Widowed	13 (4.3%)
Never married	30 (10.0%)
Age	
Mean age (SD)	31.36 years (8.2)
HIV status	
HIV-positive	59 (19.7%)
HIV-negative (from a test < 12 months ago)	172 (57.3%)
Status unknown (never tested or tested negative more than 12 months ago)	69 (23.0%)
Primary Occupation	
Fisherman	75 (25.0%)
Fishmongerer	75 (25.0%)
Alcohol Seller	75 (25.0%)
Commercial Sex Worker	75 (25.0%)
Mean Monthly Earnings (with SD)^a^	$55.82 ($51.56)
Monthly Earnings (converted to US dollars)	
≥ $120	92 (30.7%)
$61–$119	66 (22.0%)
$25–60	64 (21.3%)
< $25	78 (26.0%)

^a^converted from Ugandan shillings to US dollars

**Table 2:** Mean item scores and distribution of CES-D response options in a sample from rural Ugandan fishing communities

CES-D Scale Item (original 20-item scale)	Proportion of Participants who selected each response option.			
	0	1	2	3
1. I was bothered by things that usually don’t bother me.	0.60	0.18	0.17	0.06
2. I did not feel like eating; my appetite was poor.	0.57	0.13	0.21	0.08
3. I felt that I could not shake off the blues even with help from my family or friends	0.67	0.10	0.16	0.07
4. I felt I was just as good as other people.^a^	0.18	0.17	0.30	0.35
5. I had trouble keeping my mind on what I was doing.	0.60	0.14	0.15	0.11
6. I felt depressed.	0.38	0.24	0.22	0.17
7. I felt that everything I did was an effort.	0.17	0.26	0.27	0.30
8. I felt hopeful about the future.^a^	0.79	0.12	0.04	0.05
9. I thought my life had been a failure.	0.76	0.11	0.07	0.06
10. I felt fearful.	0.55	0.21	0.14	0.11
11. My sleep was restless.	0.53	0.19	0.17	0.10
12. I was happy.^a^	0.39	0.30	0.13	0.17
13. I talked less than usual.	0.34	0.36	0.19	0.11
14. I felt lonely.	0.42	0.33	0.13	0.12
15. People were unfriendly.	0.35	0.07	0.12	0.46
16. I enjoyed life.^a^	0.36	0.33	0.12	0.18
17. I had crying spells.	0.86	0.07	0.05	0.02
18. I felt sad.	0.48	0.28	0.14	0.10
19. I felt that people dislike me.	0.38	0.11	0.20	0.31
20. I could not get “going.”	0.63	0.18	0.11	0.08

^a^ Indicates the item was reverse scored

### Exploratory factor analysis

EFA was performed to determine the extent to which a single underlying latent variable explained the common variance among the remaining 17 scale items. First the items were viewed in a single factor structure. Applying the four criteria described in the methods section (eigen values greater than one; the proportion of the variance accounted for by the factors (> 0.3); no significant crossloading of items onto multiple factors (> 0.3); and each factor having a minimum of three items), a one-factor solution was not supported. For three items (4, 7, and 15), a one-factor solution did not satisfy all criteria and thus additional factor solutions were examined. We then fit the items to a two, three, and four factor structure. Applying our factor eligibility criteria, we removed two additional items (items 4 and 7); an insufficient proportion of the variance was accounted for by a one and two-factor structure and in a three-factor structure, the two items formed their own factor, failing to meet the minimum number of items per factor criteria. We then reran the EFA with our 15-item scale. This time, two additional items (15 and 19) did not meet eligibility criteria in a single factor structure because they did not load (> 0.3) on the primary factor. In the two and three-factor structures these items had very high communalities (h2 of 0.65 and 0.91 in the two-factor and h2 of 0.56 and 0.81 in the three-factor, for items 15 and 19, respectively) and were highly correlated with one another, but as a set, they were insufficient in number of items to group into a separate domain. Since these items also did not meet our factor inclusion criteria when fitted to a one, two, three or four factor structure, we decided to exclude them from the model as well and focus on the primary construct. This left us with a 13-item scale. We then reran the EFA a third time and this time, all items loaded onto the primary construct. We fitted the data to two, three, and four factor models and based on factor eligibility criteria, we determined that a single factor model was the best fit in our sample. Table 3 contains a list of all retained scale items (*n* = 13) and their factor loadings for the single factor model. We also ran a parallel analysis scree, which supported this conclusion. All additional analyses assume that the scale is unidimensional, measuring only one latent construct. In this single factor model, item loadings onto the latent variable ranged from 0.4–0.8. Items 1, 13 and 20 had the lowest factor loadings (loadings of 0.4, 0.5 and 0.5, respectively); the other ten items had factor loadings of 0.6 or above, with items 6, 10, 11, 14 and 18, having the highest loadings (loadings of 0.8) onto the latent trait.

**Table 3:** Factor loadings for 13 items retained in the CES-D scale

CES-D Scale Item (revised 13-item scale)	Factor Loading in single factor model
1. I was bothered by things that usually don’t bother me.	0.4
2. I did not feel like eating; my appetite was poor.	0.6
3. I felt that I could not shake off the blues even with help from my family or friends	0.6
5. I had trouble keeping my mind on what I was doing.	0.7
6. I felt depressed.	0.8
10. I felt fearful.	0.8
11. My sleep was restless.	0.8
12. I was happy.^a^	0.7
13. I talked less than usual.	0.5
14. I felt lonely.	0.8
16. I enjoyed life.^a^	0.7
18. I felt sad.	0.8
20. I could not get “going.”	0.5

^a^ indicates the item was reverse scored

### Reliability

#### Item response theory approach

**Fig. 2:**
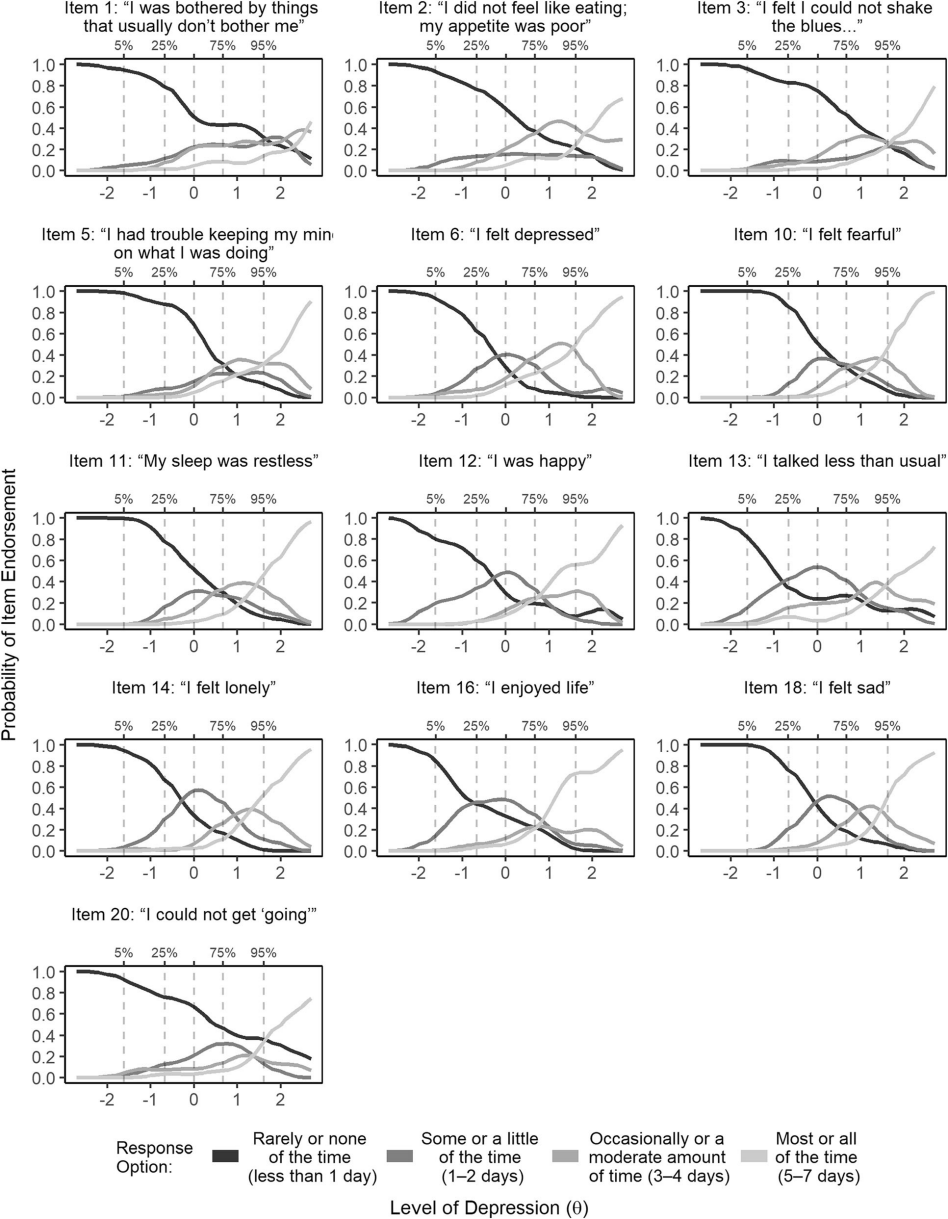
Option Characteristic Curves (OCCs) for the 13 items included in the final scale. The Y axis indicates the probability of selecting a particular response option and the X axis represents levels of the latent construct. The plotted lines represented the different response options and the dotted vertical lines within the plot areas indicate the 5th, 25th, 50th, 75th, and 95th quintiles

Following EFA, we assessed the non-parametric item response model by looking at the observed score distributions in the OCCs and the proportion of respondents who selected each response option for the remaining thirteen items (see Table 2 and Fig. 2). The OCCs show the probability of selecting each response option for an item based on an individual’s level of the latent trait. The OCC’s for the final set of items defining a single primary construct demonstrated better item performance than the seven removed items but many of the retained items only differentiated at very high levels of depression for this sample and therefore don’t provide information across lower levels of the latent trait.

Additionally, some retained items continued to show little variability in responses, with the majority of respondents endorsing a single response option for some items. For example, in item 1, “I was bothered by things that usually don’t bother me”, 60% of participants chose option 0, “rarely or none of the time”. The remaining options were only endorsed rarely in this sample and only among those above the 95th percentile for level of depression severity. Similarly, for scale item number 3, “I felt that I could not shake off the blues even with help from my family or friends, 67% of participants chose option 0 and the OCC for that item revealed that only at 1.5 SDs above the mean level of the latent trait does the probability of selecting a different response option exceed the probability of selecting option 0.

**Fig. 3:**
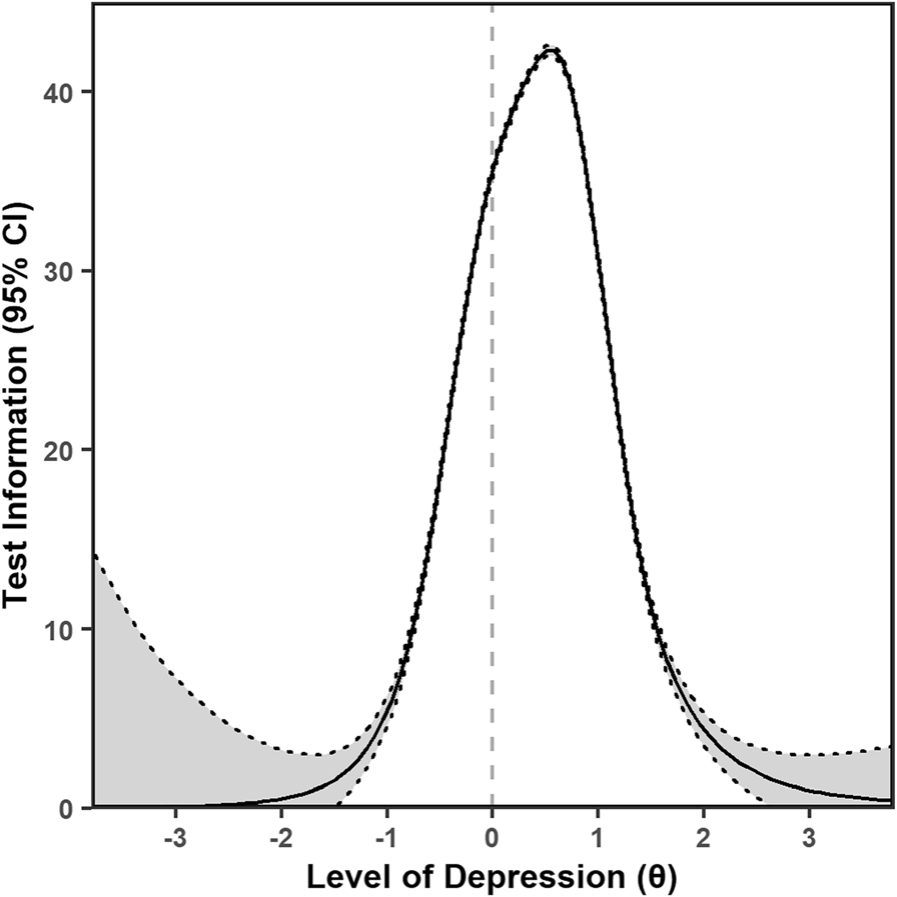
Test Information Function and Standard Error of Measurement for the reduced 13-item CES-D. The Y axis indicates the amount of information (solid line) or error (shaded area between the dotted lines) that the reduced 13 item CES-D has at various levels of the latent construct, which is represented by the X axis

We then fit the graded response model (parametric IRM) for the 13-items that formed our final set of items. The test information function was normally distributed with most of the information concentrated around the mean level of the latent trait (represented as “0” on the x-axis) in our sample and almost all information within 2 standard deviations of the mean (Fig. 3).

#### Classical test theory approach

Cronbach’s coefficient alpha is a measure of internal reliability that estimates the ratio of common source variation to total variation among the scale items. We found an overall coefficient alpha of 0.89, which suggests that the abbreviated 13-item scale had very high internal consistency in this population. We found a McDonald’s total coefficient omega of 0.91, which is slightly higher but still very similar to our Cronbach’s coefficient alpha of 0.89. We also found a McDonald’s hierarchical omega of 0.79. Hierarchical omega is based on the sum of the squared loadings onto the general factor only, so our relatively high hierarchical omega supports our decision to model a single primary dimension in our dataset.

### Validity

#### Content validity

Content Validity was assessed by grouping the 13 retained scale items into the four domains first identified by Radloff during scale development. Within each of the domains, we compared the number of retained items to the number of items in the full (20-item) scale to assess if sufficient content was retained in each domain (see Table 4). The domain with the greatest proportion of retained items was the somatic domain (six out of seven items). In the full scale, the depressed mood domain also contains seven items, five of which were retained in our 13-item scale. Half of the four items in the positive affect domain were retained and neither of the two items in the interpersonal domain were retained. The two items that comprise the interpersonal domain in the full scale were item 19 and item 15, EFA revealed that these items were highly correlated with one another (as would be expected) but did not map onto the primary construct and did not fit into a factor containing any other items. One of our eligibility criteria during EFA was that each factor include ≥3 items, so both of these items were dropped.

**Table 4:** Mean item scores, by domain, for the retained CES-D scale items in the present sample and a comparison sample

CES-D Scale Item (revised 13-item scale)	Mean Item Score (SD) from present study [*n* = 300]	Mean Item Score (SD) from NHANES (34) [*n* = 2814]
Domain 1: Depressed mood [items for this domain in original scale = 7]		
Item 3. I felt that I could not shake off the blues even with help from my family or friends	0.62 (0.98)	0.24 (0.6)
Item 10. I felt fearful.	0.81 (1.04)	0.26 (0.61)
Item 18. I felt sad.	0.85 (0.99)	0.38 (0.66)
Item 14. I felt lonely.	0.95 (1.02)	0.37 (0.74)
Item 6. I felt depressed.	1.17 (1.11)	0.44 (0.72)
Domain 2: Somatic [items for this domain in original scale = 7]		
Item 20. I could not get “going.”	0.64 (0.97)	0.52 (0.78)
Item 1. I was bothered by things that usually don’t bother me.	0.68 (0.94)	0.34 (.66)
Item 5. I had trouble keeping my mind on what I was doing.	0.78 (1.06)	0.41 (0.73)
Item 2. I did not feel like eating; my appetite was poor.	0.81 (1.04)	0.29 (0.69)
Item 11. My sleep was restless.	0.84 (1.05)	0.65 (0.89)
Item 13. I talked less than usual.	1.06 (0.98)	0.49 (0.82)
Domain 3: Positive affect^a^ [items for this domain in original scale = 4]		
Item 12. I was happy.	1.09 (1.1)	0.60 (0.95)
Item 16. I enjoyed life.	1.13 (1.1)	0.53 (0.97)
Domain 4: Interpersonal [items for this domain in original scale = 2; none retained]		

^a^ indicates items in the domain were reverse scored

#### Construct validity

After assessing the representation of content in this sample, we then assessed the numeric relationships of individual item means within the four domains to determine construct validity. This was achieved by looking at item performance within each domain to see if rank order of the means in the current sample were matched to those observed in other samples. Scores were compared within each domain to one another and also to item rank order from a large sample of US residents participating in the National Health and Nutrition Examination Survey (NHANES) [34]. For score comparisons within our own sample, we expected to see less severe items within a domain to have higher mean scores (i.e. higher frequencies endorsed by more people) than more severe items. We also expected similar items within a domain to produce similar items means. In comparing our item rank order to the rank order found by Carleton et al. in the NHANES sample, we expected to see similarities. Actual item means were not compared across the two studies because these samples, although both from the general population (as opposed to a clinical population) are from very different settings and contexts that are not comparable. Table 4 contains mean items scores, grouped by domain and listed in ascending order based on mean item score in our sample as well as the comparison group from Carleton et al.’s study.

Within the depressive mood domain, the order of items (ascending from lowest to highest mean) was nearly identical in the two studies. The item endorsed with the lowest frequency was item 3, “I couldn’t shake the blues even with the help of family or friends” and the item with the highest mean was “I felt depressed”. In the somatic domain, there was minimal variation in mean scores across the retained items in either sample (items means ranged from 0.64–1.06 in the present study sample and 0.29–0.65 in the NHANES sample). In this domain, comparison of items ranked by mean from the two samples revealed different relative relationships. The item with lowest frequency of endorsement in our study sample was Item 20 “I could not get going”, which was one of the highest-ranking items in the NHANES sample. Across domains, items had higher means in the present study sample. Item 20 is the closest mean across the two samples of any item (0.64 and 0.52 in our sample and NHANES, respectively). In the positive affect domain, only two items were retained in the 13-item scale and the rank order of these two items was not consistent across the samples. However, within both samples, the two items had nearly identical mean scores, making the discrepancy in rank order less concerning.

## Discussion

In the fishing communities along Lake Victoria, a number of items in the full 20-item scale did not perform well and this was an unexpected finding. Poor performance of individual items could be attributed to a number of reasons. It is possible that the translation of certain items obscured their meaning. During initial evaluation of item performance using the OCCs, the observed low endorsement across items 8, 9 and 17, despite relatively high scores on the cumulative scale, suggests that the option endorsements for these items were not strongly related to overall scores on the latent trait (the first option was almost always more likely than the others across the full range of observed scores and no additional options appear more likely than another, even at the highest levels of depression (>95th). These items may be too severe for use in this sample, so they were excluded prior to EFA. During EFA, an additional four items (4, 7, 15, and 19) were excluded for not meeting our factor analysis criteria which left us with a 13-item single factor scale.

Despite originally finding a four-factor structure for the CES-D, Radloff advocated for the scale to be treated as a unidimensional measure because all of the items were highly correlated with the primary construct, *depression*, even though they mapped onto four domains [19]. Because of the variability in factor structures identified in previous studies [34], we did not expect any specific factor structure to emerge during EFA but we did predict that the number of factors would not exceed four. Our prediction was confirmed and a single factor model was the best fit for our reduced 13-item scale, suggesting translational success of the retained items. In our final factor structure, the higher factor loadings (> 0.6) of some items suggests that these items were the most defining while items with lower factor loadings were less deterministic of the latent trait in our sample.

In our final scale, item performance was still sub-optimal. For example, nearly 70% of participants answered, “rarely or none of the time” to item 3: “I felt that I could not shake off the blues even with help from my family or friends”. Independent back translation of the items to English during the present paper’s analysis revealed that translators made efforts to avoid the literal translation of western idioms such as “shake the blues” (back translation of item 3, “You thought that you would not succeed even with support from your family”), but it is still possible that this translation obscured the item’s original meaning in the study context. Researchers validating a translated version of the Luganda Patient Health Questionnaire (PHQ-9) concluded that poor performance of some items could be attributed to failure to replace the existing idioms with culturally appropriate ones [38]. It is also possible that items that didn’t perform well were not relevant to depression in this context. A third possible explanation is that in the context of these rural Ugandan fishing communities, where HIV as well as income insecurity are prevalent, depressive symptoms included in the CES-D, that individuals in the United States (where the CES-D was developed, piloted and validated) may recognize as irregular, may not have been recognized by participants as something unusual (and therefore not worth mentioning). For example, not sleeping well and loss of appetite (both items on the CES-D) in this setting could be driven by financial worries, HIV, or even side effects of antiretroviral therapy.

There is evidence that the CES-D performs differently among persons living with HIV (PLWH). A recent validation study of the CES-D among PLWH in the U.S. found serious psychometric limitations when using the CES-D in this population [39]. In that study, five of the items performed poorly and were removed from scale; one of these items, item 8 “I feel hopeful about the future”, demonstrated differential item functioning by HIV status; this item also performed poorly in our sample and was excluded from our reduced scale. Gay et al.’s finding is consistent with another validation study from New Zealand that concluded that items in the positive affect domain could not differentiate between PLWH who were depressed and those who were not depressed (i.e. these items could not detect the latent trait, depression, among PLWH) [40]. Natamba et al. found substantially higher mean CES-D scores among women living with HIV compared to HIV negative women (15.3 compared to 21.5) attending an antenatal clinic in northern Uganda, but did not report which items performed differently by HIV status [23].

Regardless of the reason(s) for poor performance of items, removal of the items that were not well aligned with a primary construct, allowed us to obtain a very high internal reliability (coefficient alpha = 0.89; coefficient total omega = 0.91) with the retained items. When assessing the scale's reliability, using nonparametric items response, we were reluctant to remove the items with little variability in responses (e.g. item 3 and 1) given poor representation of response patterns at the higher levels of depression and thus inability to effectively evaluate the full item characteristics. Instead we opted retain the items since they met our a priori factor criteria. When assessing the parametric items response, the height and density of information around the mean in the test information function curve suggests that the CES-D’s precision at estimating the latent trait was unequal across abilities (severity of trait) and most precise around the mean level of the latent trait in our sample. Our measures of reliability from classical test theory, Cronbach’s alpha, McDonald’s omega both suggest high internal reliability and the high Hierarchical omega supports our decisions to use a single factor structure.

Construct validity in our sample ranged from good to moderate across the four scale domains. In the depressive domain, similarities in patterns of endorsement between our sample and the NHANES comparison sample suggest good construct validity. In the somatic domain there was a divergence in response patterns between the two samples. One possible explanation for this and the low endorsement of item 20 is that translation of this item may have obscured or changed its meaning. Despite differences in rank order of means in this domain, similar ranges for items means suggest moderate construct validity. The two retained items in the positive affect domain were not in the same rank order, but the means were similar enough across studies to suggest reasonable construct validity within this domain. No items in the interpersonal domain were retained in our reduced scale so the construct validity of that domain could not be assessed.

### Public health significance and recommendations

There is a dearth of knowledge on the epidemiology of depression in sub-Saharan Africa, generally, and specifically in Uganda. In recent years, acknowledgment of the potential public health impact of not addressing this deficit has led to increased calls to focus on assessing the burden of mental health throughout Africa as well as the availability of mental health resources [41, 42]. Much of this push has focused on the intersecting vulnerabilities of depression and HIV and the potential to avert new infections and improve treatment outcomes and quality of life among PLWH by identifying and intervening on depression [8]. To our knowledge this is the first study to validate the Luganda version of the CES-D. While a few studies have used the CES-D in Uganda [23, 25, 27], only one has validated the scale and that study was conducted using Luo dialect versions of the scale, among pregnant women [23]. Our paper contributes to the evidence base on the use of CES-D in Uganda by validating the scale in a previously unvalidated language and by applying both CCT and IRT approaches to offer a robust assessment of scale performance in a sample of men and women. Furthermore, this work was completed among a highly vulnerable population, fishing community residents, who experience a high burden of depression risk factors and co-morbidities. Establishing a locally validated CES-D for use in this setting will allow for depression prevalence estimates to be captured precisely. Accurate estimates of depression in these communities and identification of sub-populations most affected can facilitate the development and implementation of targeted mental health interventions in these communities.

Based on our findings, we recommend that future use of the CES-D in this population (fisherfolk in Uganda) use our revised version of the scale with the final set of 13-items. We suggest that the other three items with relatively poor performance in this population (due to response variability occurring only at extreme levels of the trait) be monitored in future use to see if they too should be omitted. We also recommend that those translating the CES-D into languages other than English work closely with their translators to ensure idioms such as “Feeling blue” are not directly translated and instead are replaced with more culturally appropriate idioms. Piloting of the translated items prior to study implementation and qualitative research exploring perceptions of depression and how it relates to interpersonal issues (a domain that was not represented in our reduced scale) and positive affect in this context could also shed light on why items in these two domains performed so poorly and serve as a starting point for generation of a new pool of items to be piloted as part of the scale in this setting. Evidence from other developing countries suggests that the generation of new items with local idioms, either for a new scale or to replace poorly performing items in an existing scale, can generate a more precise measure of depression [43].

Researchers utilizing the CES-D in this setting in the future should also attempt to include items that can improve the rigor of validation efforts (such as other depression measures, inclusion of a clinical depression measure and administration in both a clinical and general population sample). Given the prevalence of HIV and heavy drinking in the fishing communities, both which are associated with depression, it is also important for future studies to include large enough samples (> 200 subjects per strata) so that differential item functioning by group membership (e.g. HIV positive and negative) can be assessed.

### Limitations

This study has a number of limitations. Study design and the data collection tool precluded assessment of some forms of validity. The study questionnaire did not contain any other screening measures for depression so convergent validity could not be assessed. No diagnostic assessment of depression was conducted as part of the study, so criterion validity could not be assessed. Due to small sample size, we were unable to perform differential item functioning to look for differences in item performance by HIV status, gender or profession [44]. Providing compensation for participation, however small, could have introduced selection bias. The cross-sectional nature of the study prohibited comparison of score via test-retest validation. In addition, participants self-reported their HIV testing history and results and therefore readers should use caution when interpreting the sample description regarding HIV status. Despite these limitations, this paper is a valuable contribution to the sparse evidence base validating the Luganda version of the CES-D.

## Conclusions

In summary, this paper addresses a gap in the literature regarding the validity, reliability, and performance of the Luganda version of the CES-D as a screening tool for depression in Uganda. It provides the first validation of this measure among a sample of men and women in Uganda and is the first attempt to validate the scale in Luganda. We recommend our revised 13-item scale be used for future research in this setting. Qualitative work is needed to understand why items from certain domains performed poorly in this setting and to identify suitable local idioms to replace the poor performing items from the original scale.

## Supplementary information

**Additional file 1.** CES-D data from Wakiso Dataset. This dataset only contans the variables need to complete the psychometric analyses presented in this manuscript (i.e., the 20 items from the full CES-D scale). Reverse coded items (4, 8, 12 and 16) have already been transposed (i.e., all items are now coded in the same direction)

**Additional file 2.** Luganda version of reduced 13-item CESD scale with English back translations.

## Data Availability

The dataset supporting the conclusions of this article is included within the article (see Additional file 1: Dataset). The reduced 13-item Luganda version of the CES-D with back-translation to English is available in Additional file 2: Luganda CES-D.

## Abbreviations

CES-D: Center for Epidemiologic Studies Depression Scale
CTT: Classical Test Theory
DALY: Disability adjusted life years
EFA: Exploratory Factor Analysis
HIV: Human Immunodeficiency Virus
IRT: Item Response Theory
MDD: Major Depressive Disorder
OCC: Operating Characteristic Curve
PLWH: Persons Living with HIV
SD: Standard Deviation
