# Supplementary material for: Challenges in measuring depression among Ugandan fisherfolk: a psychometric assessment of the Luganda version of the Center for Epidemiologic Studies Depression Scale (CES-D)
Source: BMC Psychiatry. 2020 Feb 5;20:45. doi: 10.1186/s12888-020-2463-2 (PMC7003345; doi:10.1186/s12888-020-2463-2)
Supplement: Supplementary file 2 — Additional file 2. Luganda version of reduced 13-item CESD scale with English back translations. [file 12888_2020_2463_MOESM2_ESM.docx]

**Luganda version of reduced 13-item CESD scale with English back translations**

1. **Wateganyizibwa ebintu ebitatera kukuteganya**

Back translation to English: You were bothered by things that rarely bother you.

1. **Wawulira nga toyagala kulya, ng’ekilya tekiliko**

Back translation to English: You felt like you didn't want to eat, when you didn't have the appetite.

1. **Walowooza nti wali tojja kumalako wadde ng’abekikakyo bakwatiddeko.**

Back translation to English: You thought that you wouldn't succeed even with support from your family

1. **Walina obuzibu nga toterela kukyokola**

Back translation to English: You had a problem where you couldn't concentrate on something you were doing.

1. **Wanakuwala**

Back translation to English: You felt sad.

1. **Wajjula okutya**

Back translation to English: You were filled with fear.

1. **Otulo twakubula**

Back translation to English: You lacked sleep.

1. **Wali musanyufu**

Back translation to English: You were happy.

1. **Wayogera kitono okusinga bulijjo**

Back translation to English: You talked less than usual.

1. **Wawubaala**

Back translation to English: You felt lonely.

1. **Wanyumilwa obulamu**

Back translation to English: You enjoyed life.

1. **Wanakuwala**

Back translation to English: You felt sad.

1. **Wawuulira nga tolina kikuwaliriza kukola kintu kyona**

Back translation to English: You felt like you didn't have a motivation to do anything.
